# Supplementary material for: Large language models for thematic analysis in healthcare research: A blinded mixed-methods comparison with human analysts
Source: PLOS Digit Health. 2026 Apr 3;5(4):e0001189. doi: 10.1371/journal.pdig.0001189 (PMC13048440; doi:10.1371/journal.pdig.0001189)
Supplement: S1 Text — (DOCX) [file pdig.0001189.s001.docx]

Good Reporting of A Mixed Methods Study (GRAMMS) checklist

Title: Large Language Models for Thematic Analysis in Healthcare Research: A Blinded Mixed-Methods Comparison with Human Analysts

| Guideline | Section: page |
| --- | --- |
| Describe the justification for using a mixed methods approach to the research question | Methods: page 5 |
| Describe the design in terms of the purpose, priority and sequence of methods | Methods: pages 5-10 |
| Describe each method in terms of sampling, data collection and analysis | Methods pages 5-10 |
| Describe where integration has occurred, how it has occurred and who has participated in it | Methods pages 8-10 |
| Describe any limitation of one method associated with the presence of the other method | Discussion: pages 24-25 |
| Describe any insights gained from mixing or integrating methods | Discussion: pages 22-27 |

*Ref: O'Cathain A, Murphy E, Nicholl J. The quality of mixed methods studies in health services research. J Health Serv Res Policy. 2008;13: 92-98*
