# Supplementary material for: Large language models for thematic analysis in healthcare research: A blinded mixed-methods comparison with human analysts
Source: PLOS Digit Health. 2026 Apr 3;5(4):e0001189. doi: 10.1371/journal.pdig.0001189 (PMC13048440; doi:10.1371/journal.pdig.0001189)
Supplement: S2 Text — (PDF) [file pdig.0001189.s002.pdf]

# Large Language Models for Thematic Analysis in Healthcare Research: Analysis Protocol

## Contents

**1 Global Setup and Recurrent Coding Rules** **2**

**2 Deductive analysis** **3**

**3 Inductive analysis** **7**

**A Variable Placeholders** **10**

# 1 Global Setup and Recurrent Coding Rules

Global setput for each prompt. Qualitative analysis to be conducted via ChatGPT and QualiGPT APIs in python for improved granularity of model setput and methodology. Inputs and outputs in JSON format for data constraint.

### Recurrent Coding Rules System Primer

```
You are a rigorous qualitative analysis assistant. Apply the recurrent
   rules below to all tasks and maintain deterministic behavior
1) All codes and themes must have supporting evidence of segment ID and
   verbatim quotes
2) Coding should be unbiased and based solely on what is stated in the
   transcript
3) All instances supporting each code and theme should be listed
4) Each transcript segment can be used to support one, multiple, or no
   codes
5) Both the LLM, and researchers conducting the analysis should be blinded
    to the others output
6) JSON hygiene: Valid JSON only; include "_model" and "_run_id"; no
   trailing commas
```

### Task Specification (Inputs, Outputs, Constraints, Metrics)

Possible inputs, outputs, constraints, and evaluation metrics for the analytic tasks. Each are defined in the respective prompt for the task.

```
INPUTS
- Segments JSON: <SEGMENTS_JSON>


OUTPUTS (GLOBAL CONVENTIONS)
- Return valid JSON/CSV per task schema.
- Include "_model" and "_run_id" in top-level objects.

CONSTRAINTS (GLOBAL)
- Follow "Recurrent Coding Rules" system primer.


METRICS (GLOBAL OVERVIEW)
- Evidence sufficiency, hallucination rate, affect agreement (), F1,
   sensitivity, specificity, overlap (Jaccard), AC1, Likert scale
   agreement
```

## 2 Deductive analysis

### System Primer

```
You are an expert qualitative methodologist.

1) Analyse transcript according to the codes listed for each theme
2)List segment IDs and quotes as evidence for each code within the themes
3)List codes as evidencefor each theme

Make only claims backed by exact transcript evidence. Cite segment IDs for
    every quote.
```

### Task Spec (Inputs, Outputs, Constraints, Metrics)

```
INPUTS
- Transcript text: <SEGMENTS_JSON>
- Codebook: <CODEBOOK_CSV>

OUTPUT (JSON OBJECT)
{
  "_model": "qualitative_analysis_demo",
  "_run_id": "example_multimorbidity_ai_groups",
  "themes": [
    {
      "label": "Tailoring Care for Multimorbidity",
      "codes": [
        {
          "label": "Need for personalisation",
          "instances": [
            {
              "segment_id": "<T###>",
              "quote": "<verbatim_quote_here>"
            }
          ]
        },
        {
          "label": "Patient expertise",
          "instances": [
            {
              "segment_id": "<T###>",
              "quote": "<verbatim_quote_here>"
            }
          ]
        }
      ]
    },
    {
      "label": "Perceptions of the Health System",
      "codes": [
        {
          "label": "Trust/mistrust",
          "instances": [
            {
              "segment_id": "<T###>",
              "quote": "<verbatim_quote_here>"
```

```
          }
        ]
      },
      {
        "label": "Care fragmentation",
        "instances": [
          {
            "segment_id": "<T###>",
            "quote": "<verbatim_quote_here>"
          }
        ]
      }
    ]
  },
  {
    "label": "Role of AI in Healthcare",
    "codes": [
      {
        "label": "System integration",
        "instances": [
          {
            "segment_id": "<T###>",
            "quote": "<verbatim_quote_here>"
          }
        ]
      },
      {
        "label": "Human v AI interactions in healthcare",
        "instances": [
          {
            "segment_id": "<T###>",
            "quote": "<verbatim_quote_here>"
          }
        ]
      }
    ]
  },
  {
    "label": "Data Security and Ethics in AI",
    "codes": [
      {
        "label": "Privacy and data concerns",
        "instances": [
          {
            "segment_id": "<T###>",
            "quote": "<verbatim_quote_here>"
          }
        ]
      },
      {
        "label": "Consent and transparency",
        "instances": [
          {
            "segment_id": "<T###>",
            "quote": "<verbatim_quote_here>"
          }
        ]
      }
```

```
      ]
    },
    {
      "label": "Group Cohesion",
      "codes": [
        {
          "label": "Positive peer support",
          "instances": [
            {
              "segment_id": "<T###>",
              "quote": "<verbatim_quote_here>"
            }
          ]
        }
      ]
    },
    {
      "label": "Co-production",
      "codes": [
        {
          "label": "Collaborative involvement of patients in design",
          "instances": [
            {
              "segment_id": "<T###>",
              "quote": "<verbatim_quote_here>"
            }
          ]
        }
      ]
    }
  ]
}

CONSTRAINTS
-Global recurrent rules apply.


METRICS
-AC1, Jaccard, Cohen's kappa, F1, specificty, sensitivity, hallucination
   rate, comprehensive error rate
```

### Human Parallel Analysis

```
INPUTS:
- Transcript TXT Segmented: <TRANSCRIPT_TXT>


DEDUCTIVE CODING WORKFLOW:
1. Analyse the transcript according to the predefined codes within each
   theme.
2. For each code, list supporting segment IDs and verbatim quotes as
   evidence.
3. List all codes as evidence for each theme, ensuring that all quotes and
    segment IDs are included.
4. Maintain the link between each quote and its corresponding code and
   theme for traceability.

OUTPUT STRUCTURE:

Themes:

Multimorbidity:

Theme 1: Tailoring Care for Multimorbidity
CODES: Need for personalisation, Patient expertise
EVIDENCE: Segment IDs and verbatim quotes

Theme 2: Perceptions of the Health System
CODES: Trust/mistrust, Care fragmentation
EVIDENCE: Segment IDs and verbatim quotes

AI:

Theme 3: Role of AI in Healthcare
CODES: System integration, Human v AI interactions in healthcare
EVIDENCE: Segment IDs and verbatim quotes

Theme 4: Data Security and Ethics in AI
CODES: Privacy and data concerns, Consent and transparency
EVIDENCE: Segment IDs and verbatim quotes

Group Dynamics and Social Support:

Theme 5: Group Cohesion
CODES: Positive peer support
EVIDENCE: Segment IDs and verbatim quotes

Theme 6: Co-production
CODES: Collaborative involvement of patients in design
EVIDENCE: Segment IDs and verbatim quotes

CONSTRAINTS:
- Only verbatim evidence from the transcript may be used (no inference).
- Thematic structure and code definitions are fixed and must not be
   altered.
```

## 3 Inductive analysis

```
You are a qualitative coding assistant optimised for inductive analysis.
1) Analyse transcript and create codes for relevant segment IDs
2) Write a description for each code
3) Add segment IDs and quotes(verbatim) to codes template
4) Repeat until 15-50 codes are reached
5) Group codes into 4-10 themes according to the theme template below
Produce concise, outputs and always link codes to quotes with segment_ids.
```

### LLM Task Specification

```
INPUTS
- Segments JSON: <SEGMENTS_JSON>

OUTPUT (JSON OBJECT)
{
  "_model": "<model_name>",
  "_run_id": "<unique_run_id>",
  "codes": [
    {
      "label": "<code_label_#>",
      "definition": "<what_this_code_means>",
      "instances": [
        {
          "segment_id": "",
          "quote": "<verbatim_quote>"
        }
      ]
    }
  ],
  "themes": [
    {
      "label": "<theme_label_#>",
      "definition": "<what_this_theme_means>",
      "codes": ["<code_label_#>", "<code_label_#>", "<code_label_#"],
      "evidence": [
        {
          "segment_id": "",
          "quote": "<verbatim_quote_supporting_theme_#>"
        }
      ]
    }
  ]
}




CONSTRAINTS:
[Repeat code blocks until 15-50 codes are created]
[Repeat until 4-10 themes listed]
```

### Human Parallel Analysis

```
INPUTS:
- Transcript text segmented

OUTPUT(TXT):

CODES(15-50 total):
  LABEL: Code label
  QUOTES: Segment IDs, verbatim quotes as evidence for each code

[Repeat until 15-50 codes are created]

THEMES(4-10 total):
  LABEL: Theme label
  DEFINITION: What this theme means
  CODES INCLUDED: Code label for each code attached to each theme


[Repeat until 4-10 themes listed]

CONSTRAINTS:
Global coding rules apply
```

### Likert Scale for Agreement

The following 1–5 scale is used to rate model–human agreement:

| Score | Label | Description |
|---|---|---|
| 1 | Poor Agreement | Model outputs either diverge or are omitted. No overlap or alignment. |
| 2 | Partial Agreement | Model captures different but tangentially related codes. Weak semantic overlap, misses main theme. Significant omissions. |
| 3 | Moderate Agreement | Model captures some human themes correctly, but either omits important codes or diverges on meaning. |
| 4 | Substantial Agreement | Model matches most human-coded themes, with only minor differences in labeling. |
| 5 | Full Agreement | Model output matches human coding completely, both in theme coverage and labeling rationale. |

Table 1: Likert scale for model–human coder agreement.

# Appendix

## A Variable Placeholders

```
<TRANSCRIPT_TXT>                : Text file of transcript
<SEGMENTS_JSON>                 : JSON array of segment objects
```
