# Supplementary material for: Large language models for thematic analysis in healthcare research: A blinded mixed-methods comparison with human analysts
Source: PLOS Digit Health. 2026 Apr 3;5(4):e0001189. doi: 10.1371/journal.pdig.0001189 (PMC13048440; doi:10.1371/journal.pdig.0001189)
Supplement: Attachment — Submitted filename: Response to Reviewers.pdf [file pdig.0001189.s007.pdf]

February 14, 2026

Editor, PLOS Digital Health

Dear Editor,

We thank you and the reviewers for the careful and constructive feedback on our manuscript, *"Large Language Models for Thematic Analysis in Healthcare Research: A Blinded Mixed-Methods Comparison with Human Analysts"*. The primary revisions to the manuscript focus on improving methodological transparency and clarifying interpretation of the results.

Below, we address each reviewer comment and summarise the corresponding revisions.

Response to Reviewer 1

Comment 1: This is a strong and well-written manuscript.

Response: We thank the reviewer for this feedback.

Comment 2: Introduction: In situating the proposed research within prior work, consider providing a thematic or theoretical synthesis beyond a descriptive synthesis.

Response: We agree with the reviewer that the existing literature could be more clearly synthesised in the manuscript.

Manuscript changes:

- **Introduction:** Added paragraph focused on synthesising the existing literature.

Comment 3: Consider strengthening the justification of methodological choices, e.g., the epistemological assumptions behind the use of expert adjudication as a reference model, non-inferiority margins, and the treatment of Likert-scale as interval data.

Response: We have expanded upon these methodological choices in the methods section.

Manuscript changes:

- **Methods:** Explained use of expert adjudication model and implied assumptions.
- **Methods:** Justified conducting a non-inferiority analysis and non-inferiority margins more clearly.
- **Methods:** Justified the use of the Likert scale as interval data.

Response to Reviewer 2

Comment 1: Clarify the description, characteristics, and institutional affiliation of the adjudication panel and coders, and how that may influence the results.

Response: We have clarified the institutional affiliation of the panel and coders, and its implications for interpretation of results.

Manuscript changes:

- **Methods (Reflexivity):** Noted explicitly the institutional affiliation and background of researchers and the potential impact of researcher's background on results.
- **Discussion(Strengths and Limitations):** Acknowledged the potential impact of researcher's background on results.

Comment 2: Sample size is insufficient to comment on inter-human agreement

Response: We agree that phrasing on inter-human agreement could be refined.

Manuscript changes:

- **Throughout manuscript:** Tempered language on inter-human agreement.

Comment 3: Discuss how the study's limitations may affect its interpretation

Manuscript changes:

- **Discussion (Strengths and Limitations):** Discussed interpretation of results contextually
- **Discussion (Strengths and Limitations):** Explicitly acknowledged that inductive analysis results should be treated with caution due to lower number of data points.

Comment 4: Clarify the nature of the dataset and focus group sessions more clearly

Manuscript changes:

- **Methods:** Added additional detail on the nature of the focus group and facilitation

Other minor revision suggestions: Improve figure readability, define PPI, and correct typo.

Manuscript changes:

- **Throughout manuscript** The figures have been revised for readability. PPI has now been defined in the introduction, and the typo has been corrected.

Response to Reviewer 3

Comment 1: This is an interesting, well-designed mixed-methods comparison.

Response: We thank the reviewer for this feedback.

Comment 2: The use of a single focus group limits generalisability. Discuss the impact of topic familiarity and topic area in reference to LLM training data

Response: We thank the reviewer for this comment and agree that this is an important point of discussion.

Manuscript changes:

- **Discussion and Limitations:** We discuss the potential impact of topic choice and its relation to our findings given that healthcare is a widely discussed topic.

Comment 3: Provide more detail on the adjudication process, including resolution of disagreements and metrics of initial inter-rater reliability.

Response: We thank the reviewer for this feedback and agree that reporting initial inter-rater reliability would strengthen the manuscript. We have described the adjudication process in more detail, including how the initial codes were derived and resolution of disagreements. Inter-rater reliability metrics are now reported.

Manuscript changes:

- **Methods:** Clarified adjudication and initial coding process.
- **Methods:** Reported inter-rater reliability metrics

Comment 4: Discuss relative importance of error-metrics or consider a weighted error metric

Response: We agree that this could be explored in more detail in the manuscript, and we have now expanded the result verification section to address this.

Manuscript changes:

- **Results (Result verification):** Discussed potential practical impact of errors in thematic analysis

Comment 5: Clarify implications of process-orientated framing of LLM analysis

Response: We agree that this point could be clarified and discussed further in the manuscript.

Manuscript changes:

- **Discussion:** Discussed our reported process-orientated framing, and its potential effect depending on research context.

Comments 6 and 7: Discuss practical implications of the results for integration of LLMs in qualitative analysis workflows, including suitability, cost-effectiveness and decision-workflows.

Response: We thank the reviewer for these suggestions. We now include a decision framework in the conclusions, suggesting where LLM integration may be more or less appropriate based on the results of our study.

As analysis time was not formally measured, we cannot draw firm conclusions regarding efficiency or cost-effectiveness. However, we suggest that quantification of this is an area of potential future study. We also discuss the potential efficiency improvements as well as additional time taken for LLM verification.

Manuscript changes:

- **Discussion:** Discussed practical implications of implementation, including efficiency and verification time, as well as suggesting this as an area of future study.
- **Discussion (Conclusion):** Suggested a decision framework for the use of LLMs in thematic analysis grounded in the results of our study

We thank the reviewers again for their feedback, which we believe has improved the manuscript's framing, transparency, and methodological clarity.

Yours sincerely,

Callum Hill (On behalf of all co-authors)
Primary Care Research Centre
Aldermoor Health Centre,
University of Southampton
Southampton, SO16 5ST, UK
Email: ch1g22@soton.ac.uk
